# Structural and luminescent properties of co-crystals of tetra­iodo­ethyl­ene with two aza­phenanthrenes

**DOI:** 10.1107/S2056989020002182

**Published:** 2020-02-25

**Authors:** Yu-Jin Cui, Feng Su, Wei-Jun Jin

**Affiliations:** a Department of Chemistry, Changzhi University, Changzhi 046011, People’s Republic of China; b Key Laboratory Chemical Biology and Molecular Engineering, Education Ministry, People’s Republic of China

**Keywords:** co-crystallized mol­ecules, crystal structure, tetra­iodo­ethyl­ene, aza­phenanthrenes, luminescence

## Abstract

Two new co-crystals, tetra­iodo­ethyl­ene–phenanthridine (1/2), 0.5C_2_I_4_·C_13_H_9_N (**1**) and tetra­iodo­ethyl­ene–benzo[*f*]quinoline (1/2), 0.5C_2_I_4_·C_13_H_9_N (**2**), were obtained from tetra­iodo­ethyl­ene and aza­phenanthrenes, and characterized by IR and fluorescence spectroscopy, elemental analysis and X-ray crystallography. In the crystal structures, C—I⋯*π* and C—I⋯N halogen bonds link the independent mol­ecules into one-dimensional chains and two-dimensional networks with subloops. In addition, the planar aza­phenanthrenes lend themselves to *π*–*π* stacking and C—H⋯*π* inter­actions, leading to a diversity of supra­molecular three-dimensional structural motifs being formed by these inter­actions. Luminescence studies show that co-crystals **1** and **2** exhibit distinctly different luminescence properties in the solid state at room temperature.

## Abstract 2

Two new co-crystals of tetra­iodo­ethyl­ene with two aza­phenanthrenes were successfully synthesized. In the crystals, C—I⋯*π* and C—I⋯N halogen bonds link the mol­ecules. A study of their luminescence properties indicates that co-crystals **1** and **2** exhibit distinctly different luminescence in the solid state at room temperature.

## Chemical context   

A halogen bond is an attractive non-covalent inter­action between an electrophilic region in a covalently bonded halogen atom and a Lewis base. Halogen bonding (XB) is a powerful tool to assemble supra­molecular materials and to promote chemical or biological mol­ecular recognition (Desiraju *et al.*, 2013 ▸; Cavallo *et al.*, 2016 ▸; Gilday *et al.*, 2015 ▸; Wang *et al.*, 2016 ▸). Over the past few years, XB has been used successfully to assemble luminescent co-crystals (Liu *et al.*, 2017*a* ▸; d’Agostino *et al.*, 2015 ▸; Ventura *et al.*, 2014 ▸; Bolton *et al.*, 2011 ▸). XB can play multiple roles in co-crystals, for example, as cement to assemble XB donors and acceptors together (Metrangolo *et al.*, 2005 ▸), and, importantly, as a heavy-atom source to enhance phospho­rescence or delayed fluorescence by efficient spin-orbital coupling (Gao *et al.*, 2012 ▸). Phospho­rescence or delayed fluorescence materials are very popular for preparing light devices because of the higher inter­nal quantum efficiency of triplet excitons (Brown *et al.*, 1993 ▸; Baldo *et al.*, 1999 ▸).
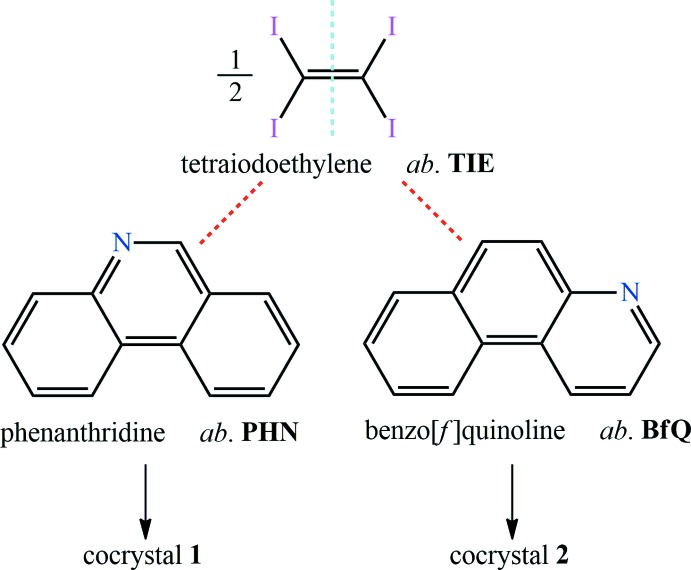


Nitro­gen heteroaromatic rings are a common type of luminescence or luminescent precursor materials. However, in general, it is difficult to use them to generate phospho­rescence or delayed fluorescence. Haloperfluoro­benzenes, as XB donors, have been used in attempts to assemble luminescence co-crystals with aza­phenanthrenes (Gao *et al.*, 2017 ▸; Wang *et al.*, 2014 ▸, 2016 ▸; Wang & Jin, 2017 ▸; Liu *et al.*, 2017*b* ▸). We report herein the use of tetra­iodo­ethyl­ene (TIE) as a new XB donor in the assembly of co-crystals with two different aza­phenanthrenes, namely phenanthridine (PHN) and benzo[*f*]quinoline (BfQ), which is expected to tune their luminescence behaviour *via* a change of the co-crystal structures. Single crystal X-ray diffraction (XRD) data reveal that the two co-crystals of TIE with PHN and BfQ reported here have inter­esting structural properties and exhibit different luminescence behaviour from previous reports. TIE as a quadridentate XB donor allows the formation of three-dimensional halogen-bonded networks with XB acceptors, PHN and BfQ. Using the conventional solution-based method, yellow co-crystals suitable for XRD measurement were obtained. The crystal structures of the co-crystals are mainly constructed by C—I⋯*π* and C—I⋯N halogen bonds. Other multiple inter­molecular inter­actions, such as *π*–*π* stacking, C—H⋯*π*, C—H⋯I as well as C—H⋯H—C inter­actions, are also observed in the co-crystals.

## Structural commentary   

**Figure 1:**
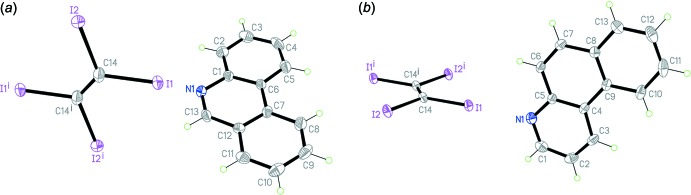
The mol­ecular structures of co-crystals **1** and **2**, showing the atom-labelling scheme and displacement ellipsoids at the 30% probability level [Symmetry codes: (i) −*x* + 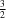, −*y* + 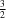, −*z* + 1 for co-crystal **1**; (i) −*x*, −*y* + 1, −*z* + 2 for co-crystal **2**].

The asymmetric units of co-crystals **1** and **2** each comprise one half TIE mol­ecule lying about an inversion centre and one PHN or BfQ mol­ecule in a general position, hence the co-crystals have a 1:2 stoichiometry (Fig. 1 ▸). Co-crystal **1** crystallizes in the monoclinic space group *C*2/*c* while **2** crystallizes in the triclinic space group *P* 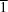.

## Supra­molecular features   

**Figure 2:**
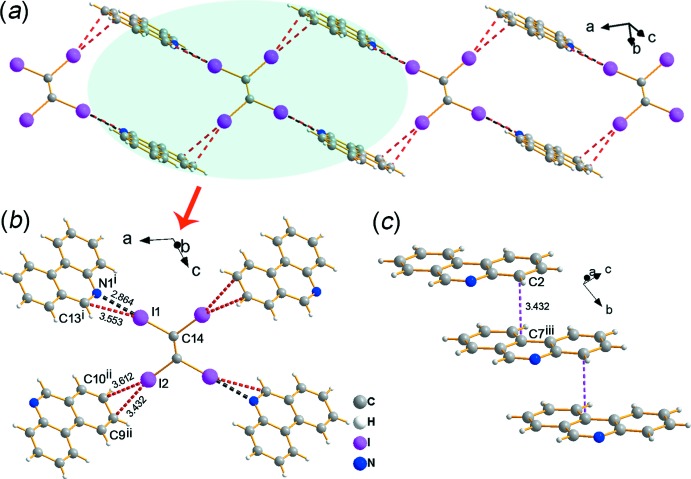
Crystal packing of **1**. (*a*) The two-dimensional network structure formed by C—I⋯N, C—I⋯C and C—I⋯π halogen bonds. (*b*) The structural motif extracted from the two-dimensional network. (*c*) The π–π stacking inter­actions extracted from the three-dimensional structure.

**Figure 3:**
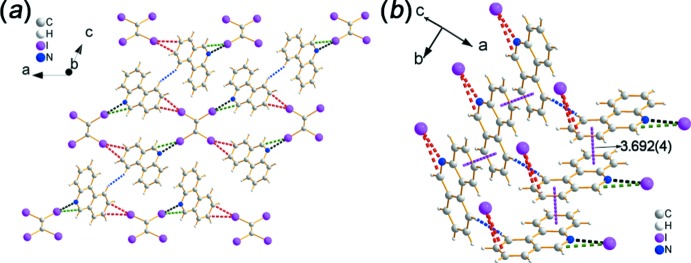
Crystal packing of **1**. (*a*) The two-dimensional network extends along the *a*- and *c*-axis directions, and two directions connect by C—H⋯H—C inter­actions. (*b*) The two-dimensional networks connected by π–π stacking and C—H⋯H—C inter­actions to form a three-dimensional structure.

In the crystal of **1**, C—I⋯N, C—I⋯C and C—I⋯*π* halogen bonds lead to the formation of a two-dimensional network structure in which the rectangular motif has a *D*⋯2*A*⋯*D*⋯2*A* arrangement, as shown in Fig. 2 ▸ *a*. The I1 atom of the TIE mol­ecule inter­acts with the N1 atom of a PHN mol­ecule, forming a C1—I1⋯N1 halogen bond (Fig. 2 ▸ *b*). The I1⋯N1^i^ distance is 2.864 (7) Å and the corresponding C14–I1⋯N1^i^ angle is 172.8 (2)° [symmetry code: (i) *x*, *y* + 1, *z*]. The strong C14—I1⋯N1 halogen bond results in a I1⋯C13^i^ distance [3.553 (8) Å] shorter than the sum of the van der Waals radii, which indicates a C1—I1⋯C13 halogen inter­action. In addition, the C14—I2⋯C9^ii^/C10^ii^ C—I⋯*π* separations [Fig. 2 ▸ *b*; symmetry code: (ii) 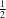 − *x*, 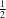 − *y*, *z*] are 3.432 (9) and 3.612 (8) Å, and the corresponding bond angles are 165.6 (2) and 156.7 (2)°, respectively. Furthermore, *π*–*π* stacking [Fig. 2 ▸ *c*; *Cg*1⋯*Cg*2^iii^ = 3.692 (4) Å, *Cg*3⋯*Cg*2^iii^ = 3.626 (4) Å; *Cg*1, *Cg*2 and *Cg*3 are the centroids of rings C1–C6, C7–C12 and N1/C1/C6/C7/C12/C13, respectively; symmetry code: (iii) *x*, −1 + *y*, *z*] and C—H⋯H—C inter­actions between two adjacent PHN mol­ecules contribute to the extension of the two-dimensional network into a three-dimensional supra­molecular structure (Fig. 3 ▸).

**Figure 4:**
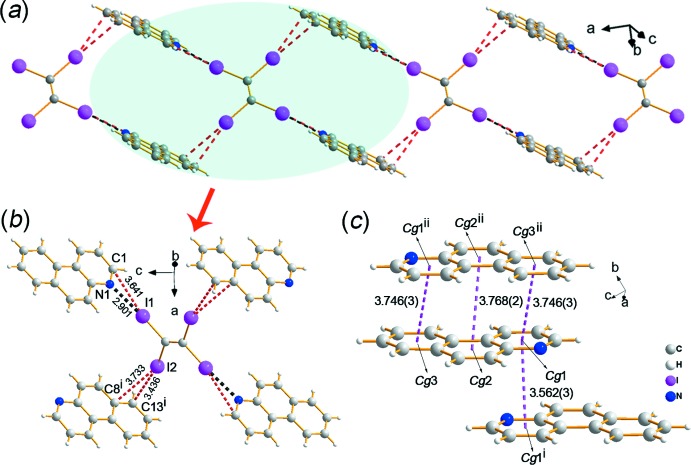
Crystal packing of **2**. (*a*) The two-dimensional network structure formed by C—I⋯N and C—I⋯π halogen bonds. (*b*) The structural motif of the two-dimensional network. (*c*) The π–π stacking inter­actions in the three-dimensional structure.

**Figure 5:**
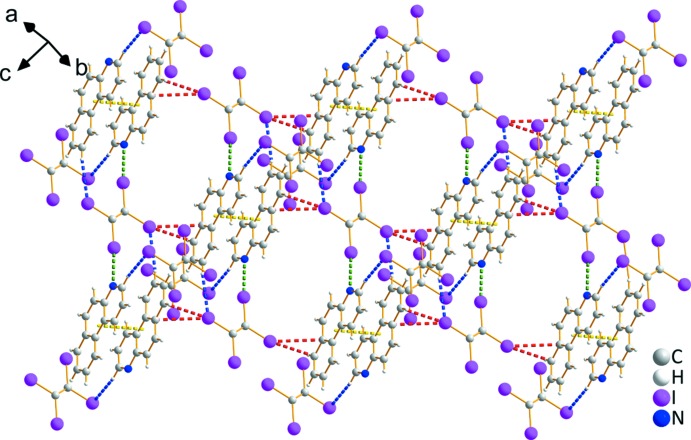
Crystal packing of **2**. The two-dimensional network extends along two directions, by C—I2⋯H7 inter­actions and π–π stacking in one direction, and by C—I2⋯H1 inter­actions and π–π stacking in the other.

The two-dimensional network of co-crystal **2** is similar to that of **1**, as shown in Fig. 4 ▸ *a*. Both of them are constructed by the same halogen-bonded synthon, *i.e*., C—I⋯N, C—I⋯C and C—I⋯*π* halogen bonds, but the bonding characteristics are slightly different. In general, the distances of the C—I⋯N, C—I⋯C and C—I⋯*π* inter­actions [I1⋯N1 = 2.901 (4), I1⋯C1 = 3.641 (5), I2⋯C13(−*x*, 1 − *y*, 1 − *z*) = 3.436 (5) and I2⋯C8(−*x*, 1 − *y*, 1 − *z*) = 3.733 (4) Å, respectively] in co-crystal **2** are all a little longer (0.004–0.121 Å) than in **1** (Fig. 4 ▸ *b*). In addition, the two-dimensional network (Fig. 5 ▸) is extended to a three-dimensional supra­molecular structure by *π*–*π* stacking (Fig. 4 ▸ *c* and 5; *Cg*1⋯*Cg*1^i^ = 3.562 (3) Å, *Cg*1⋯*Cg2* ^ii^ = 3.963 (2) Å, *Cg*1⋯*Cg3* ^ii^ = 3.746 (3) Å, *Cg*2⋯*Cg*2^ii^ = 3.768 (2) Å; *Cg*1, *Cg*2 and *Cg*3 are the centroids of rings N1/C1–C5, C4–C9 and C8–C13, respectively; symmetry codes: (i) 1 − *x*, 1 − *y*, 1 − *z*; (ii) 1 − *x*, −*y*, 1 − *z*] and C—H⋯I hydrogen bonds (Table 1 ▸).

## Powder X-ray diffraction pattern   

**Figure 6:**
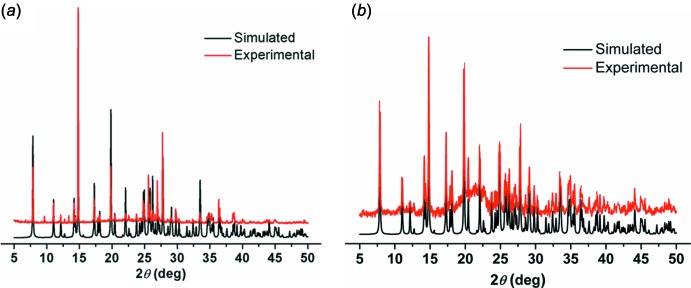
Powder X-ray diffraction pattern of co-crystals (*a*) **1** and (*b*) **2**.

The powder X-ray diffraction (PXRD) experiments were carried out for the title co-crystals using a Bruker D8-ADVANCE X-ray diffractometer (Cu *K*α, λ = 1.5418 Å) in the 2θ range of 5 to 50°. As shown in Fig. 6 ▸, the experimental patterns for **1** and **2** match well with the spectra simulated from the XRD data, which confirms the purity of **1** and **2**.

## Luminescence behavior of co-crystals 1 and 2   

**Figure 7:**
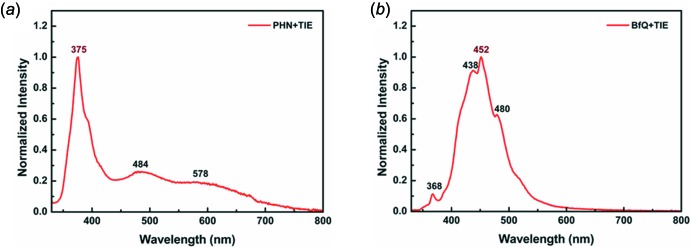
Total luminescence spectra of co-crystals (*a*) **1** and (*b*) **2** (excitation at 300 nm) measured under fluorescence mode.

**Figure 8:**
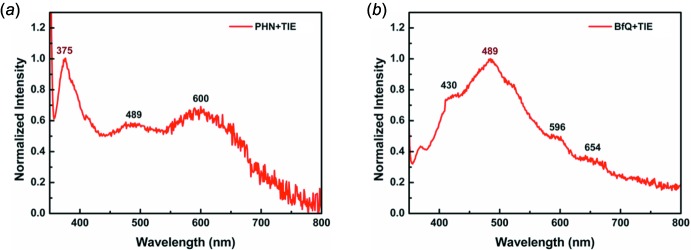
Luminescence spectra of co-crystals (*a*) **1** and (*b*) **2** (excitation at 330 nm) measured under phospho­rescence mode.

As shown in Fig. 7 ▸, the two co-crystals fluoresce with some vibrational fine structure (see also spectroscopic data in Table 2 ▸). The two co-crystals also show delayed fluorescence (Fig. 8 ▸). For both co-crystals, the emission bands in the region of 450–480 nm should be relative to the *π*–*π* stacking patterns. Luminescence from the excimer is possible because of the close *π*–*π* stacking distances as shown in Figs. 2 ▸–5 ▸ ▸ ▸, besides luminescence from a monomer. Furthermore, TIE–PHN and TIE–BfQ produce weak phospho­rescence. The strong XB inter­action between the iodine atoms of TIE and the non-bonding orbitals of the aza­phenanthrene N atoms should cause the energy of the lowest ^1^(*n*, π*) state to drop below that of the ^3^(*π*, π*) state. It is supposed that for the singlet states the 0–0 transition of emitters in co-crystals is localized at 375 nm and 450 nm, respectively, and for triplet states the 0–0 transition is at about 600 nm. The energy gap between *S*_1_ and *T*_1_ is largely greater than 20 kJ mol^−1^, so the delayed fluorescence most likely originates from the triplet–triplet annihil­ation process, named *P*-type delayed fluorescence (P-DF). Both delayed fluorescence and phospho­rescence are relative to triplet states, so they should be significant for improving the exciton emission efficiency of luminescence materials (Adachi *et al.*, 2001 ▸).

For the luminescence decay, all singlet state decay lifetimes (11.49 ns for **1** and 9.29 ns for **2**) are about 10 ns, while the delayed fluorescence lifetime (4.36 µs for **1** and 6.45 µs for **2**) is less than the 10 µs level because of the strong heavy-atom effect leading to a faster decay of the triplet state. Additionally, the phospho­rescence is too weak to measure its decay lifetime. However, the phospho­rescence lifetime can be estimated to be about 20 µs based on the relationship between P-DF and the accompanying phospho­rescence (Parker *et al.*, 1962 ▸, 1965 ▸).

## Synthesis and crystallization   

0.1 mmol of PHN/BfQ and 0.05 mmol of TIE were dissolved in an acetone/chloro­form (2:1) mixture in a glass vial. Well–formed co-crystals **1** and **2** suitable for single-crystal X–ray diffraction (XRD) measurements were obtained by slow evaporation of the solvent at room temperature after about two weeks. Elemental analysis (%, EA) calculated for C_14_H_9_NI_2_ (445.02): C 37.78, H 2.04, N 3.15. Found: C 37.54, H 2.31, N 3.26. For co-crystal **1**, and C 37.85, H 2.16, N 3.04 for co-crystal **2**. IR (KBr, ν, cm^−1^) For **1**: 3048(*w*), 1603(*w*), 1572(*w*), 1494(*m*), 1446(*w*), 1382(*m*), 1293(*m*), 1267(*m*), 1189(*m*), 1089(*m*), 948(*m*), 870(*s*), 832(*s*), 802(*s*), 749(*s*), 707(*s*), 615(*m*), 538(*m*), 487(*m*), 435(*m*). For **2**: 3048(*w*), 1611(*w*), 1576(*s*), 1522(*w*), 1486(*w*), 1458(*m*), 1440(*m*), 1238(*m*), 1132(*m*), 1032(*m*), 953(*m*), 924(*m*), 889(*s*), 745(*s*), 714(*s*), 610(*m*), 552(*m*), 448(*m*), 423(*m*).

## Refinement   

Crystal data, data collection and structure refinement details are summarized in Table 3 ▸. H atoms attached to C atoms were positioned geometrically and refined as riding on their parent atoms, with C—H = 0.93 Å and *U*_iso_(H) = 1.2*U*_eq_(C).

## Supplementary Material

Crystal structure: contains datablock(s) 1, 2. DOI: 10.1107/S2056989020002182/vm2225sup1.cif

Structure factors: contains datablock(s) 1. DOI: 10.1107/S2056989020002182/vm22251sup4.hkl

Structure factors: contains datablock(s) 2. DOI: 10.1107/S2056989020002182/vm22252sup5.hkl

CCDC references: 1899078, 1899077

Additional supporting information:  crystallographic information; 3D view; checkCIF report

## Figures and Tables

**Table 1:** Hydrogen-bond geometry (Å, °) for (2)

*D*—H⋯*A*	*D*—H	H⋯*A*	*D*⋯*A*	*D*—H⋯*A*
C1—H1⋯I2^i^	0.93	3.16	4.019 (5)	155
C6—H6⋯I1	0.93	3.31	3.945 (4)	127

Symmetry code: (i) 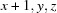.

**Table 2:** Phospho­rescent characteristics of co-crystals at room temperature

		**TIE–PHN**	**TIE–BfQ**
Total luminescent spectra	λ_ex_/nm	300	300
	λ_em_ /nm	375, 484, 578	368, 438, 452, 480
	τ_average_/ ns	11.49	9.29
			
DF and phospho­rescent spectra	λ_ex_/nm	330	330
	λ_em_ /nm	375, 489, 600	430, 489, 596, 654
	τ_average_/ µs	4.36	6.45

**Table 3:** Experimental details

	**1**	**2**
Crystal data		
Chemical formula	0.5C_2_I_4_·C_13_H_9_N	0.5C_2_I_4_·C_13_H_9_N
*M*_r_	445.02	445.02
Crystal system, space group	Monoclinic, *C*2/*c*	Triclinic, *P* 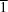
Temperature (K)	296	296
*a*, *b*, *c* (Å)	24.2920 (18), 4.8348 (4), 24.8761 (16)	7.3179 (4), 8.1089 (5), 11.3252 (7)
α, β, γ (°)	90, 116.272 (2), 90	97.050 (2), 92.059 (2), 95.579 (2)
*V* (Å^3^)	2619.8 (3)	663.02 (7)
*Z*	8	2
Radiation type	Mo *K*α	Mo *K*α
μ (mm^−1^)	4.78	4.72
Crystal size (mm)	0.30 × 0.25 × 0.25	0.35 × 0.32 × 0.30

Data collection		
Diffractometer	Bruker APEXII CCD	Bruker APEXII CCD
Absorption correction	Multi-scan (*SADABS*; Bruker, 2012 ▸)	Multi-scan (*SADABS*; Bruker, 2012 ▸)
*T*_min_, *T*_max_	0.489, 0.745	0.638, 0.745
No. of measured, independent and observed [*I* > 2σ(*I*)] reflections	12094, 2635, 1782	8567, 2701, 2099
*R*_int_	0.045	0.026
(sin θ/λ)_max_ (Å^−1^)	0.624	0.625

Refinement		
*R*[*F* ^2^ > 2σ(*F* ^2^)], *wR*(*F* ^2^), *S*	0.043, 0.078, 1.03	0.028, 0.058, 1.08
No. of reflections	2635	2701
No. of parameters	154	154
H-atom treatment	H-atom parameters constrained	H-atom parameters constrained
Δρ_max_, Δρ_min_ (e Å^−3^)	1.08, −0.62	0.80, −0.74

Computer programs: *APEX2* and *SAINT* (Bruker, 2012 ▸), *SHELXS97* and *SHELXTL* (Sheldrick, 2008 ▸), *SHELXL2018/3* (Sheldrick, 2015 ▸), (Sheldrick, 2008 ▸), *DIAMOND* (Brandenburg, 2005 ▸) and *publCIF* (Westrip, 2010 ▸).

## supplementary crystallographic information

### Tetraiodoethylene–phenanthridine (1/2) (1) Crystal data

**Table:**

0.5C_2_I_4_·C_13_H_9_N	*F*(000) = 1648
*M**_r_* = 445.02	*D*_x_ = 2.257 Mg m^−^^3^
Monoclinic, *C*2/*c*	Mo *K*α radiation, λ = 0.71073 Å
*a* = 24.2920 (18) Å	Cell parameters from 5311 reflections
*b* = 4.8348 (4) Å	θ = 3.1–26.3°
*c* = 24.8761 (16) Å	µ = 4.78 mm^−^^1^
β = 116.272 (2)°	*T* = 296 K
*V* = 2619.8 (3) Å^3^	Block, yellow
*Z* = 8	0.30 × 0.25 × 0.25 mm

### Tetraiodoethylene–phenanthridine (1/2) (1) Data collection

**Table:**

Bruker APEXII CCD diffractometer	1782 reflections with *I* > 2σ(*I*)
Radiation source: sealed tube	*R*_int_ = 0.045
φ and ω scans	θ_max_ = 26.3°, θ_min_ = 3.1°
Absorption correction: multi-scan (*SADABS*; Bruker, 2012)	*h* = −30→29
*T*_min_ = 0.489, *T*_max_ = 0.745	*k* = −6→6
12094 measured reflections	*l* = −31→30
2635 independent reflections	

### Tetraiodoethylene–phenanthridine (1/2) (1) Refinement

**Table:**

Refinement on *F*^2^	0 restraints
Least-squares matrix: full	Hydrogen site location: inferred from neighbouring sites
*R*[*F*^2^ > 2σ(*F*^2^)] = 0.043	H-atom parameters constrained
*wR*(*F*^2^) = 0.078	*w* = 1/[σ^2^(*F*_o_^2^) + (0.0187*P*)^2^ + 28.9715*P*] where *P* = (*F*_o_^2^ + 2*F*_c_^2^)/3
*S* = 1.03	(Δ/σ)_max_ = 0.001
2635 reflections	Δρ_max_ = 1.08 e Å^−^^3^
154 parameters	Δρ_min_ = −0.61 e Å^−^^3^

### Tetraiodoethylene–phenanthridine (1/2) (1) Special details

**Table:**

Geometry. All esds (except the esd in the dihedral angle between two l.s. planes) are estimated using the full covariance matrix. The cell esds are taken into account individually in the estimation of esds in distances, angles and torsion angles; correlations between esds in cell parameters are only used when they are defined by crystal symmetry. An approximate (isotropic) treatment of cell esds is used for estimating esds involving l.s. planes.

### Tetraiodoethylene–phenanthridine (1/2) (1) Fractional atomic coordinates and isotropic or equivalent isotropic displacement parameters (Å^2^)

**Table:**

	*x*	*y*	*z*	*U*_iso_*/*U*_eq_	
I1	0.70990 (2)	0.95088 (10)	0.57060 (2)	0.04011 (16)	
I2	0.85225 (2)	0.96131 (11)	0.56611 (2)	0.04316 (16)	
N1	0.6417 (3)	0.1927 (12)	0.6276 (2)	0.0377 (14)	
C1	0.6440 (3)	0.1453 (14)	0.6836 (3)	0.0339 (16)	
C2	0.6849 (3)	−0.0548 (15)	0.7193 (3)	0.0442 (18)	
H2	0.708323	−0.152775	0.704671	0.053*	
C3	0.6910 (4)	−0.1093 (16)	0.7755 (3)	0.053 (2)	
H3	0.719345	−0.239978	0.799494	0.064*	
C4	0.6547 (4)	0.0319 (17)	0.7965 (3)	0.054 (2)	
H4	0.658318	−0.006933	0.834537	0.065*	
C5	0.6141 (3)	0.2253 (16)	0.7623 (3)	0.0452 (19)	
H5	0.590382	0.318582	0.777344	0.054*	
C6	0.6070 (3)	0.2880 (14)	0.7044 (3)	0.0328 (16)	
C7	0.5640 (3)	0.4901 (13)	0.6650 (3)	0.0342 (16)	
C8	0.5240 (3)	0.6464 (16)	0.6801 (3)	0.0475 (19)	
H8	0.524161	0.622630	0.717264	0.057*	
C9	0.4853 (3)	0.8313 (17)	0.6406 (4)	0.054 (2)	
H9	0.459174	0.933001	0.651309	0.065*	
C10	0.4834 (3)	0.8741 (16)	0.5846 (4)	0.051 (2)	
H10	0.456128	1.001451	0.558148	0.061*	
C11	0.5219 (3)	0.7271 (15)	0.5691 (3)	0.0455 (19)	
H11	0.521208	0.755053	0.531834	0.055*	
C12	0.5627 (3)	0.5329 (14)	0.6091 (3)	0.0345 (16)	
C13	0.6035 (3)	0.3779 (15)	0.5941 (3)	0.0405 (18)	
H13	0.602707	0.412409	0.557015	0.049*	
C14	0.7603 (3)	0.8233 (15)	0.5244 (3)	0.0411 (18)	

### Tetraiodoethylene–phenanthridine (1/2) (1) Atomic displacement parameters (Å^2^)

**Table:**

	*U*^11^	*U*^22^	*U*^33^	*U*^12^	*U*^13^	*U*^23^
I1	0.0411 (3)	0.0521 (3)	0.0327 (3)	0.0057 (2)	0.0213 (2)	−0.0022 (2)
I2	0.0332 (3)	0.0582 (3)	0.0364 (3)	−0.0048 (2)	0.0139 (2)	−0.0059 (2)
N1	0.034 (3)	0.046 (4)	0.037 (3)	0.000 (3)	0.019 (3)	−0.004 (3)
C1	0.034 (4)	0.036 (4)	0.033 (4)	−0.005 (3)	0.016 (3)	−0.005 (3)
C2	0.042 (4)	0.043 (4)	0.046 (4)	0.001 (4)	0.018 (4)	0.001 (4)
C3	0.060 (5)	0.049 (5)	0.041 (5)	0.000 (4)	0.013 (4)	0.011 (4)
C4	0.067 (6)	0.066 (6)	0.027 (4)	−0.015 (5)	0.019 (4)	−0.001 (4)
C5	0.048 (5)	0.053 (5)	0.041 (4)	−0.010 (4)	0.026 (4)	−0.008 (4)
C6	0.031 (4)	0.038 (4)	0.030 (4)	−0.008 (3)	0.014 (3)	−0.007 (3)
C7	0.032 (4)	0.034 (4)	0.041 (4)	−0.006 (3)	0.020 (3)	−0.008 (3)
C8	0.045 (5)	0.053 (5)	0.049 (5)	−0.002 (4)	0.026 (4)	−0.005 (4)
C9	0.031 (5)	0.051 (5)	0.081 (6)	−0.003 (4)	0.025 (4)	−0.015 (5)
C10	0.038 (5)	0.040 (5)	0.070 (6)	0.006 (4)	0.019 (4)	0.005 (4)
C11	0.042 (5)	0.049 (5)	0.039 (4)	−0.012 (4)	0.012 (4)	−0.003 (4)
C12	0.030 (4)	0.036 (4)	0.033 (4)	−0.003 (3)	0.010 (3)	−0.001 (3)
C13	0.046 (5)	0.052 (5)	0.030 (4)	−0.005 (4)	0.023 (4)	−0.002 (4)
C14	0.045 (5)	0.044 (5)	0.043 (4)	0.007 (4)	0.028 (4)	0.003 (3)

### Tetraiodoethylene–phenanthridine (1/2) (1) Geometric parameters (Å, º)

**Table:**

I1—C14	2.108 (6)	C6—C7	1.449 (9)
I2—C14	2.111 (7)	C7—C12	1.394 (9)
N1—C13	1.293 (8)	C7—C8	1.406 (9)
N1—C1	1.390 (8)	C8—C9	1.353 (10)
C1—C2	1.389 (9)	C8—H8	0.9300
C1—C6	1.400 (9)	C9—C10	1.387 (11)
C2—C3	1.364 (10)	C9—H9	0.9300
C2—H2	0.9300	C10—C11	1.360 (10)
C3—C4	1.389 (11)	C10—H10	0.9300
C3—H3	0.9300	C11—C12	1.408 (9)
C4—C5	1.352 (10)	C11—H11	0.9300
C4—H4	0.9300	C12—C13	1.416 (9)
C5—C6	1.406 (9)	C13—H13	0.9300
C5—H5	0.9300	C14—C14^i^	1.301 (13)
			
C13—N1—C1	117.3 (6)	C9—C8—C7	120.1 (7)
N1—C1—C2	117.1 (6)	C9—C8—H8	120.0
N1—C1—C6	122.8 (6)	C7—C8—H8	120.0
C2—C1—C6	120.1 (6)	C8—C9—C10	122.1 (7)
C3—C2—C1	120.8 (7)	C8—C9—H9	119.0
C3—C2—H2	119.6	C10—C9—H9	119.0
C1—C2—H2	119.6	C11—C10—C9	119.0 (7)
C2—C3—C4	119.4 (7)	C11—C10—H10	120.5
C2—C3—H3	120.3	C9—C10—H10	120.5
C4—C3—H3	120.3	C10—C11—C12	120.4 (7)
C5—C4—C3	120.8 (7)	C10—C11—H11	119.8
C5—C4—H4	119.6	C12—C11—H11	119.8
C3—C4—H4	119.6	C7—C12—C11	120.1 (6)
C4—C5—C6	121.2 (7)	C7—C12—C13	118.4 (6)
C4—C5—H5	119.4	C11—C12—C13	121.6 (6)
C6—C5—H5	119.4	N1—C13—C12	125.7 (6)
C1—C6—C5	117.7 (6)	N1—C13—H13	117.1
C1—C6—C7	118.1 (6)	C12—C13—H13	117.1
C5—C6—C7	124.2 (6)	C14^i^—C14—I2	121.2 (7)
C12—C7—C8	118.3 (7)	C14^i^—C14—I1	126.2 (7)
C12—C7—C6	117.7 (6)	I2—C14—I1	112.5 (3)
C8—C7—C6	124.0 (6)		
			
C13—N1—C1—C2	179.7 (6)	C5—C6—C7—C8	−0.3 (11)
C13—N1—C1—C6	0.2 (10)	C12—C7—C8—C9	0.5 (11)
N1—C1—C2—C3	178.4 (7)	C6—C7—C8—C9	179.9 (7)
C6—C1—C2—C3	−2.1 (11)	C7—C8—C9—C10	0.0 (12)
C1—C2—C3—C4	1.8 (12)	C8—C9—C10—C11	−0.5 (12)
C2—C3—C4—C5	−1.0 (12)	C9—C10—C11—C12	0.5 (11)
C3—C4—C5—C6	0.5 (12)	C8—C7—C12—C11	−0.5 (10)
N1—C1—C6—C5	−179.0 (6)	C6—C7—C12—C11	−179.9 (6)
C2—C1—C6—C5	1.5 (10)	C8—C7—C12—C13	179.3 (6)
N1—C1—C6—C7	1.0 (10)	C6—C7—C12—C13	−0.2 (10)
C2—C1—C6—C7	−178.5 (6)	C10—C11—C12—C7	0.0 (10)
C4—C5—C6—C1	−0.7 (10)	C10—C11—C12—C13	−179.7 (7)
C4—C5—C6—C7	179.3 (7)	C1—N1—C13—C12	−1.4 (10)
C1—C6—C7—C12	−0.9 (9)	C7—C12—C13—N1	1.5 (11)
C5—C6—C7—C12	179.1 (7)	C11—C12—C13—N1	−178.8 (7)
C1—C6—C7—C8	179.7 (6)		

Symmetry code: (i) −*x*+3/2, −*y*+3/2, −*z*+1.

### Tetraiodoethylene–benzo[f]quinoline (1/2) (2) Crystal data

**Table:**

0.5C_2_I_4_·C_13_H_9_N	*Z* = 2
*M**_r_* = 445.02	*F*(000) = 412
Triclinic, *P*1	*D*_x_ = 2.229 Mg m^−^^3^
*a* = 7.3179 (4) Å	Mo *K*α radiation, λ = 0.71073 Å
*b* = 8.1089 (5) Å	Cell parameters from 4699 reflections
*c* = 11.3252 (7) Å	θ = 2.8–26.4°
α = 97.050 (2)°	µ = 4.72 mm^−^^1^
β = 92.059 (2)°	*T* = 296 K
γ = 95.579 (2)°	Block, yellow
*V* = 663.02 (7) Å^3^	0.35 × 0.32 × 0.30 mm

### Tetraiodoethylene–benzo[f]quinoline (1/2) (2) Data collection

**Table:**

Bruker APEXII CCD diffractometer	2099 reflections with *I* > 2σ(*I*)
Radiation source: sealed tube	*R*_int_ = 0.026
φ and ω scans	θ_max_ = 26.4°, θ_min_ = 2.5°
Absorption correction: multi-scan (*SADABS*; Bruker, 2012)	*h* = −9→9
*T*_min_ = 0.638, *T*_max_ = 0.745	*k* = −10→10
8567 measured reflections	*l* = −14→14
2701 independent reflections	

### Tetraiodoethylene–benzo[f]quinoline (1/2) (2) Refinement

**Table:**

Refinement on *F*^2^	0 restraints
Least-squares matrix: full	Hydrogen site location: inferred from neighbouring sites
*R*[*F*^2^ > 2σ(*F*^2^)] = 0.028	H-atom parameters constrained
*wR*(*F*^2^) = 0.058	*w* = 1/[σ^2^(*F*_o_^2^) + (0.0189*P*)^2^ + 0.7413*P*] where *P* = (*F*_o_^2^ + 2*F*_c_^2^)/3
*S* = 1.08	(Δ/σ)_max_ = 0.001
2701 reflections	Δρ_max_ = 0.80 e Å^−^^3^
154 parameters	Δρ_min_ = −0.74 e Å^−^^3^

### Tetraiodoethylene–benzo[f]quinoline (1/2) (2) Special details

**Table:**

Geometry. All esds (except the esd in the dihedral angle between two l.s. planes) are estimated using the full covariance matrix. The cell esds are taken into account individually in the estimation of esds in distances, angles and torsion angles; correlations between esds in cell parameters are only used when they are defined by crystal symmetry. An approximate (isotropic) treatment of cell esds is used for estimating esds involving l.s. planes.

### Tetraiodoethylene–benzo[f]quinoline (1/2) (2) Fractional atomic coordinates and isotropic or equivalent isotropic displacement parameters (Å^2^)

**Table:**

	*x*	*y*	*z*	*U*_iso_*/*U*_eq_	
I1	0.20988 (4)	0.45307 (3)	0.82595 (2)	0.04767 (10)	
I2	−0.08934 (4)	0.75374 (3)	0.91391 (3)	0.05167 (11)	
N1	0.4565 (5)	0.3596 (5)	0.6400 (3)	0.0518 (9)	
C1	0.6267 (7)	0.4270 (6)	0.6615 (4)	0.0597 (13)	
H1	0.655445	0.495377	0.733212	0.072*	
C2	0.7655 (7)	0.4022 (6)	0.5841 (5)	0.0613 (13)	
H2	0.883874	0.453358	0.603495	0.074*	
C3	0.7274 (6)	0.3026 (6)	0.4794 (5)	0.0541 (12)	
H3	0.819507	0.286190	0.425958	0.065*	
C4	0.5492 (5)	0.2240 (5)	0.4514 (4)	0.0399 (10)	
C5	0.4171 (5)	0.2587 (5)	0.5350 (4)	0.0389 (9)	
C6	0.2327 (6)	0.1869 (5)	0.5119 (4)	0.0470 (11)	
H6	0.144788	0.211154	0.566982	0.056*	
C7	0.1844 (6)	0.0845 (5)	0.4114 (4)	0.0471 (11)	
H7	0.062732	0.038787	0.398340	0.057*	
C8	0.3128 (6)	0.0431 (5)	0.3238 (4)	0.0424 (10)	
C9	0.4960 (6)	0.1144 (5)	0.3424 (4)	0.0418 (10)	
C10	0.6199 (7)	0.0731 (6)	0.2544 (4)	0.0561 (12)	
H10	0.741510	0.119847	0.264207	0.067*	
C11	0.5643 (9)	−0.0348 (7)	0.1546 (5)	0.0730 (16)	
H11	0.648615	−0.060734	0.097457	0.088*	
C12	0.3842 (9)	−0.1063 (7)	0.1373 (4)	0.0716 (16)	
H12	0.347907	−0.180218	0.069304	0.086*	
C13	0.2612 (7)	−0.0676 (6)	0.2203 (4)	0.0555 (12)	
H13	0.140145	−0.115380	0.208420	0.067*	
C14	0.0238 (6)	0.5360 (5)	0.9536 (4)	0.0449 (10)	

### Tetraiodoethylene–benzo[f]quinoline (1/2) (2) Atomic displacement parameters (Å^2^)

**Table:**

	*U*^11^	*U*^22^	*U*^33^	*U*^12^	*U*^13^	*U*^23^
I1	0.0579 (2)	0.04585 (18)	0.04064 (16)	0.01385 (14)	0.01030 (14)	0.00193 (12)
I2	0.0700 (2)	0.04158 (18)	0.04756 (18)	0.02041 (15)	0.00980 (15)	0.00849 (13)
N1	0.058 (3)	0.051 (2)	0.049 (2)	0.0117 (19)	0.003 (2)	0.0083 (18)
C1	0.068 (3)	0.052 (3)	0.058 (3)	0.007 (3)	−0.016 (3)	0.006 (2)
C2	0.042 (3)	0.067 (3)	0.076 (4)	−0.002 (2)	−0.010 (3)	0.024 (3)
C3	0.039 (3)	0.055 (3)	0.073 (3)	0.006 (2)	0.013 (2)	0.026 (3)
C4	0.036 (2)	0.041 (2)	0.049 (2)	0.0109 (18)	0.008 (2)	0.0221 (19)
C5	0.041 (2)	0.036 (2)	0.043 (2)	0.0108 (18)	0.0065 (19)	0.0135 (18)
C6	0.042 (2)	0.054 (3)	0.048 (3)	0.008 (2)	0.015 (2)	0.009 (2)
C7	0.036 (2)	0.051 (3)	0.056 (3)	0.003 (2)	0.006 (2)	0.016 (2)
C8	0.051 (3)	0.036 (2)	0.044 (2)	0.0087 (19)	0.006 (2)	0.0144 (18)
C9	0.046 (2)	0.042 (2)	0.044 (2)	0.0161 (19)	0.014 (2)	0.0199 (19)
C10	0.059 (3)	0.066 (3)	0.052 (3)	0.023 (2)	0.021 (2)	0.022 (2)
C11	0.099 (5)	0.080 (4)	0.051 (3)	0.043 (4)	0.031 (3)	0.018 (3)
C12	0.115 (5)	0.059 (3)	0.044 (3)	0.025 (3)	0.007 (3)	0.004 (2)
C13	0.072 (3)	0.047 (3)	0.049 (3)	0.009 (2)	−0.003 (3)	0.010 (2)
C14	0.054 (3)	0.039 (2)	0.041 (2)	0.011 (2)	0.002 (2)	−0.0017 (18)

### Tetraiodoethylene–benzo[f]quinoline (1/2) (2) Geometric parameters (Å, º)

**Table:**

I1—C14	2.116 (4)	C6—H6	0.9300
I2—C14	2.111 (4)	C7—C8	1.423 (6)
N1—C1	1.313 (6)	C7—H7	0.9300
N1—C5	1.363 (5)	C8—C13	1.402 (6)
C1—C2	1.379 (7)	C8—C9	1.405 (6)
C1—H1	0.9300	C9—C10	1.403 (6)
C2—C3	1.354 (7)	C10—C11	1.365 (7)
C2—H2	0.9300	C10—H10	0.9300
C3—C4	1.402 (6)	C11—C12	1.384 (8)
C3—H3	0.9300	C11—H11	0.9300
C4—C5	1.401 (5)	C12—C13	1.355 (7)
C4—C9	1.448 (6)	C12—H12	0.9300
C5—C6	1.418 (6)	C13—H13	0.9300
C6—C7	1.339 (6)	C14—C14^i^	1.305 (8)
			
C1—N1—C5	117.4 (4)	C13—C8—C9	119.4 (4)
N1—C1—C2	123.8 (5)	C13—C8—C7	121.7 (4)
N1—C1—H1	118.1	C9—C8—C7	118.9 (4)
C2—C1—H1	118.1	C10—C9—C8	118.0 (4)
C3—C2—C1	119.2 (4)	C10—C9—C4	123.0 (4)
C3—C2—H2	120.4	C8—C9—C4	119.0 (4)
C1—C2—H2	120.4	C11—C10—C9	120.9 (5)
C2—C3—C4	120.1 (4)	C11—C10—H10	119.6
C2—C3—H3	120.0	C9—C10—H10	119.6
C4—C3—H3	120.0	C10—C11—C12	121.0 (5)
C3—C4—C5	116.5 (4)	C10—C11—H11	119.5
C3—C4—C9	124.0 (4)	C12—C11—H11	119.5
C5—C4—C9	119.5 (4)	C13—C12—C11	119.5 (5)
N1—C5—C4	123.0 (4)	C13—C12—H12	120.3
N1—C5—C6	117.1 (4)	C11—C12—H12	120.3
C4—C5—C6	119.9 (4)	C12—C13—C8	121.3 (5)
C7—C6—C5	120.4 (4)	C12—C13—H13	119.4
C7—C6—H6	119.8	C8—C13—H13	119.4
C5—C6—H6	119.8	C14^i^—C14—I2	121.4 (4)
C6—C7—C8	122.3 (4)	C14^i^—C14—I1	126.2 (4)
C6—C7—H7	118.9	I2—C14—I1	112.33 (18)
C8—C7—H7	118.9		
			
C5—N1—C1—C2	0.5 (7)	C13—C8—C9—C10	1.1 (6)
N1—C1—C2—C3	−0.3 (8)	C7—C8—C9—C10	−179.4 (4)
C1—C2—C3—C4	−0.9 (7)	C13—C8—C9—C4	−177.9 (4)
C2—C3—C4—C5	1.7 (6)	C7—C8—C9—C4	1.6 (6)
C2—C3—C4—C9	−179.4 (4)	C3—C4—C9—C10	1.3 (6)
C1—N1—C5—C4	0.5 (6)	C5—C4—C9—C10	−179.8 (4)
C1—N1—C5—C6	−179.7 (4)	C3—C4—C9—C8	−179.7 (4)
C3—C4—C5—N1	−1.6 (6)	C5—C4—C9—C8	−0.9 (6)
C9—C4—C5—N1	179.5 (4)	C8—C9—C10—C11	−0.9 (6)
C3—C4—C5—C6	178.6 (4)	C4—C9—C10—C11	178.1 (4)
C9—C4—C5—C6	−0.3 (6)	C9—C10—C11—C12	0.1 (8)
N1—C5—C6—C7	−179.0 (4)	C10—C11—C12—C13	0.4 (8)
C4—C5—C6—C7	0.8 (6)	C11—C12—C13—C8	−0.2 (7)
C5—C6—C7—C8	−0.1 (7)	C9—C8—C13—C12	−0.5 (6)
C6—C7—C8—C13	178.4 (4)	C7—C8—C13—C12	180.0 (4)
C6—C7—C8—C9	−1.1 (6)		

Symmetry code: (i) −*x*, −*y*+1, −*z*+2.

### Tetraiodoethylene–benzo[f]quinoline (1/2) (2) Hydrogen-bond geometry (Å, º)

**Table:**

*D*—H···*A*	*D*—H	H···*A*	*D*···*A*	*D*—H···*A*
C1—H1···I2^ii^	0.93	3.16	4.019 (5)	155
C6—H6···I1	0.93	3.31	3.945 (4)	127

Symmetry code: (ii) *x*+1, *y*, *z*.

## References

[bb1] Adachi, C., Baldo, M. A., Thompson, M. E. & Forrest, S. R. (2001). *J. Appl. Phys.* **90**, 5048–5051.

[bb2] Agostino, S. d’, Grepioni, F., Braga, D. & Ventura, B. (2015). *Cryst. Growth Des.* **15**, 2039–2045.

[bb3] Baldo, M. A., O’Brien, D. F., Thompson, M. E. & Forrest, S. R. (1999). *Phys. Rev. B*, **60**, 14422–14428.

[bb4] Bolton, O., Lee, K., Kim, H. J., Lin, K. Y. & Kim, J. (2011). *Nat. Chem.* **3**, 205–210. 10.1038/nchem.984 21336325

[bb5] Brandenburg, K. (2005). *DIAMOND*. Crystal Impact GbR, Bonn, Germany.

[bb6] Brown, A. R., Pichler, K., Greenham, N. C., Bradley, D. D. C., Friend, R. H. & Holmes, A. B. (1993). *Chem. Phys. Lett.* **210**, 61–66.

[bb7] Bruker (2012). *APEX2*, *SAINT* and *SADABS*. Bruker AXS Inc., Madison, Wisconsin, USA.

[bb8] Cavallo, G., Metrangolo, P., Milani, R., Pilati, T., Priimagi, A., Resnati, G. & Terraneo, G. (2016). *Chem. Rev.* **116**, 2478–2601. 10.1021/acs.chemrev.5b00484 PMC4768247 26812185

[bb9] Desiraju, G. R., Ho, P. S., Kloo, L., Legon, A. C., Marquardt, R., Metrangolo, P., Politzer, P., Resnati, G. & Rissanen, K. (2013). *Pure Appl. Chem.* **85**, 1711–1713.

[bb10] Gao, H. Y., Shen, Q. J., Zhao, X. R., Yan, X. Q., Pang, X. & Jin, W. J. (2012). *J. Mater. Chem.* **22**, 5336–5343.

[bb11] Gao, Y. J., Li, C., Liu, R. & Jin, W. J. (2017). *Spectrochim. Acta A*, **173**, 792–799. 10.1016/j.saa.2016.10.038 27810770

[bb12] Gilday, L. C., Robinson, S. W., Barendt, T. A., Langton, M. J., Mullaney, B. R. & Beer, P. D. (2015). *Chem. Rev.* **115**, 7118–7195. 10.1021/cr500674c 26165273

[bb13] Liu, R., Gao, Y. J. & Jin, W. J. (2017*a*). *Acta Cryst.* B**73**, 247–254. 10.1107/S205252061700292X 28362289

[bb14] Liu, R., Wang, H. & Jin, W. J. (2017*b*). *Cryst. Growth Des.* **17**, 3331–3337.

[bb15] Metrangolo, P., Neukirch, H., Pilati, T. & Resnati, G. (2005). *Acc. Chem. Res.* **38**, 386–395. 10.1021/ar0400995 15895976

[bb16] Parker, C. A. & Hatchard, C. G. (1962). *Proc. Roy. Soc. A*, **269**, 574–584.

[bb17] Parker, C. A., Hatchard, C. G. & Joyce, T. A. (1965). *Nature*, **205**, 1282–1284.

[bb18] Sheldrick, G. M. (2008). *Acta Cryst.* A**64**, 112–122. 10.1107/S0108767307043930 18156677

[bb19] Sheldrick, G. M. (2015). *Acta Cryst.* C**71**, 3–8.

[bb20] Ventura, B., Bertocco, A., Braga, D., Catalano, L., d’Agostino, S., Grepioni, F. & Taddei, P. (2014). *J. Phys. Chem. C*, **118**, 18646–18658.

[bb21] Wang, H., Hu, R. X., Pang, X., Gao, H. Y. & Jin, W. J. (2014). *CrystEngComm*, **16**, 7942–7948.

[bb22] Wang, H. & Jin, W. J. (2017). *Acta Cryst.* B**73**, 210–216. 10.1107/S2052520617002918 28362284

[bb23] Wang, H., Wang, W. & Jin, W. J. (2016). *Chem. Rev.* **116**, 5072–5104. 10.1021/acs.chemrev.5b00527 26886515

[bb24] Westrip, S. P. (2010). *J. Appl. Cryst.* **43**, 920–925.

